# The Efficacy of Curcumin-Mediated Photodynamic Therapy in the Treatment of Oral Squamous Cell Carcinoma: A Systematic Review of In Vitro Studies

**DOI:** 10.3390/life15060924

**Published:** 2025-06-07

**Authors:** Magdalena Kubizna, Jakub Fiegler-Rudol, Wojciech Niemczyk, Rafał Wiench

**Affiliations:** 1 Department of Oral Surgery, Faculty of Medical Sciences in Zabrze, Medical University of Silesia, 40-055 Katowice, Poland; 2 Department of Periodontal Diseases and Oral Mucosa Diseases, Faculty of Medical Sciences in Zabrze, Medical University of Silesia, 40-055 Katowice, Poland; s88998@365.sum.edu.pl (J.F.-R.); s81190@365.sum.edu.pl (W.N.); rwiench@sum.edu.pl (R.W.)

**Keywords:** curcumin, oral squamous cell carcinoma, photodynamic therapy, systematic review

## Abstract

Curcumin-mediated photodynamic therapy (PDT) has emerged as a promising approach for targeting oral squamous cell carcinoma (OSCC), a malignancy with a rising incidence. This systematic review synthesizes evidence from in vitro studies evaluating the anticancer efficacy of curcumin as a photosensitizer in PDT against OSCC cells. A comprehensive literature search across four databases identified eight eligible studies published between 2009 and 2024. The findings demonstrated that curcumin-PDT reduces OSCC cell viability, induces apoptosis, and impairs metabolic activity, particularly when curcumin is delivered via nanocarriers and activated with light sources near its absorption peak (430–457 nm). Despite methodological heterogeneity across cell lines, curcumin formulations, and light parameters, the photodynamic effects were reproducible and showed low dark toxicity. However, the lack of standardized protocols and absence of in vivo or clinical validation limit translational potential. Further preclinical research is needed to optimize treatment conditions and assess safety and efficacy in biological systems that more closely resemble the clinical environment.

## 1. Introduction

Oral squamous cell carcinoma (OSCC) is the most prevalent malignancy originating from the squamous epithelium of the oral cavity and ranks as the 16th most common cancer globally [1,2]. It constitutes approximately 90% of all oral cancers, frequently affecting the tongue, lips, and floor of the mouth [3]. The disease is often diagnosed at an advanced stage due to its rapid and aggressive progression, asymptomatic onset, and early metastatic potential, resulting in a low 5-year survival rate of 39–43% [4]. A concerning trend is the rising incidence of OSCC among younger individuals in Europe. The combined use of alcohol and tobacco remains the dominant risk factor, responsible for 70–80% of cases. However, human papillomavirus (HPV) infection and betel quid chewing (especially in Southeast Asia) are also recognized as independent risk factors. Notably, a growing number of OSCC cases now occur in individuals without a significant history of tobacco or alcohol use, suggesting the involvement of unidentified or emerging risk factors [5]. The multifactorial etiology of OSCC presents challenges for effective treatment. Standard therapeutic approaches include surgical excision, often supplemented with platinum-based chemotherapy and radiotherapy. These multimodal regimens are associated with considerable side effects—such as pain, dysphagia, salivary dysfunction, and disfigurement—which adversely affect quality of life [6]. Despite aggressive treatment, many patients exhibit limited therapeutic response, underscoring the urgent need for novel and more effective therapeutic strategies [1,2]. Photodynamic therapy (PDT) is a minimally invasive modality increasingly used across dermatology, oncology, gynecology, and urology. It involves the administration of a photosensitizer (PS) that selectively accumulates in diseased tissues, followed by targeted irradiation with light of a specific wavelength. This triggers photochemical reactions that induce localized cytotoxicity and tissue destruction [6,7,8,9,10,11,12,13,14,15]. Curcumin, a polyphenolic compound derived from turmeric, exhibits diverse biological activities, including anti-inflammatory, antioxidant, antimicrobial, antifungal, and anticancer effects [16,17]. It modulates membrane integrity and protein function, and promotes fibroblast activation, angiogenesis, collagen deposition, and epithelial regeneration—favorable properties for tissue repair and tumor targeting [18]. Importantly, curcumin absorbs blue light (with an excitation peak at 425 nm and emission around 530 nm), enabling its use as a photosensitizer in PDT [2]. However, its clinical application is hampered by poor water solubility, rapid degradation, and low systemic bioavailability [19]. These challenges can be mitigated by dissolving curcumin in solvents such as 10% dimethyl sulfoxide (DMSO), ethanol, or propylene glycol [20]. Due to its extended conjugated system, curcumin can be effectively photoactivated within the 300–500 nm range, producing significant phototoxic effects at micromolar concentrations [21]. This systematic review aims to evaluate existing in vitro research on the therapeutic potential of curcumin-mediated photodynamic therapy in the treatment of OSCC.

## 2. Materials and Methods

### 2.1. Focused Question

A systematic review was conducted according to the PICO model, formulating the research question as follows: in in vitro models of oral squamous cell carcinoma (Population) does treatment with curcumin-mediated photodynamic therapy (Intervention) lead to their destruction or more effective elimination (Outcome) as compared to light irradiation alone, curcumin as a photosensitizer, or other pharmacological treatments (Comparison)?

### 2.2. Information Sources and Search Strategy

The review was conducted in accordance with the PRISMA 2020 (Preferred Reporting Items for Systematic Reviews and Meta-Analyses) guidelines [22]. It was registered with PROSPERO (ID: CRD42024629102). The literature search was conducted from the 20th to the 22nd of March 2025. PubMed/Medline, Cochrane Library, Embase and Scopus databases were electronically searched. The following MeSH (medical subject headings), keywords, and their combinations were used for the search: (OSCC OR oral squamous cell carcinoma OR oral cancer) AND (photodynamic therapy OR PDT OR photodynamic inactivation OR PDI) AND (curcumin). Two authors conducted the searches individually using the same search terms. Then, the authors applied additional electronic filters, selecting only articles published in English, between 1 January 2009 and 31 December 2024. After searching and initially selecting potential studies for review, the authors jointly assessed the titles and abstracts of these articles to verify that they met all inclusion criteria. In the next step, to collate the data from the included studies, they conducted a collaborative full-text search to identify relevant information. Additionally, the authors conducted a snowball search, searching the reference lists of publications that were deemed suitable for full-text review.

### 2.3. Study Selection

The study hypothesis was that photodynamic therapy with curcumin could effectively reduce oral squamous cell carcinoma cells and could be an adjunct or alternative treatment for OSCC compared to traditional pharmacological therapies. The eligibility and exclusion criteria for articles in this review are presented in Table 1.

### 2.4. Risk of Bias in Individual Studies

At the initial stage of study selection, each reviewer independently assessed titles and abstracts to minimize the risk of bias. Cohen’s test [23] was used to quantify the degree of agreement between reviewers. Any disagreements regarding the decision to include or exclude a study were discussed by the authors until agreement was reached.

### 2.5. Quality Assessment and Risk of Bias Across Studies

Three reviewers conducted independent screening reviews to assess the quality of the studies, using criteria based on the presence of key information regarding photodynamic therapy and the objectivity and verifiability of the results. The risk of bias was assessed by counting the number of “yes” or “no” responses assigned to each study for the following questions: whether a specific concentration of photosensitizer was present, if the origin of the photosensitizer was stated, if an incubation time was given, whether light source parameters (type, wavelength, output power, luminous flux, and power density) were provided, if the powermeter was used, if clinical cultures of oral squamous cell carcinoma cells were used, whether a negative control group was included, if numerical results (statistics) were reported, and whether there was any missing result data. The collected data were analyzed and the classification was based on the sum of “yes” answers given to these questions. The degree of deviation was calculated based on the following point limits: high risk (0–3), moderate risk (4–6), and low risk (7–9). Results were calculated for individual studies, and an overall estimated risk of bias (low, moderate, high) was determined for each included study according to the guidelines in the Cochrane Handbook for Systematic Reviews of Interventions [14].

### 2.6. Data Extraction

Once consensus was reached on the inclusion of eligible articles, the reviewers proceeded with comprehensive data extraction, collecting detailed information such as the citation details (first author, country, and year of publication), study design, and the specific type of Oral Squamous Cell Carcinoma examined. They also recorded characteristics of the experimental and control groups, follow-up duration, and reported outcomes. Technical specifications of the light sources used—such as wavelength, fluence, power output, irradiation time, and spot size—were documented alongside details on curcumin, including its concentration, origin, and form of application. Furthermore, the reviewers noted whether nanocarriers or additional substances were employed, as well as the incubation time prior to light exposure. A meta-analysis was not performed due to the substantial methodological heterogeneity among the included studies. This included variations in cell lines, curcumin formulations (e.g., free compound vs. nanoparticle-encapsulated), light source parameters (wavelength, fluence, power), incubation protocols, and outcome measures, which precluded meaningful quantitative synthesis.

## 3. Results

### 3.1. Primary Outcome

The primary objective of this systematic review was to assess the effectiveness of curcumin-mediated photodynamic therapy in treating oral squamous cell carcinoma cells and to analyze the methodologies used in the studies.

### 3.2. Study Selection During Full-Text Analysis

**Figure 1:**
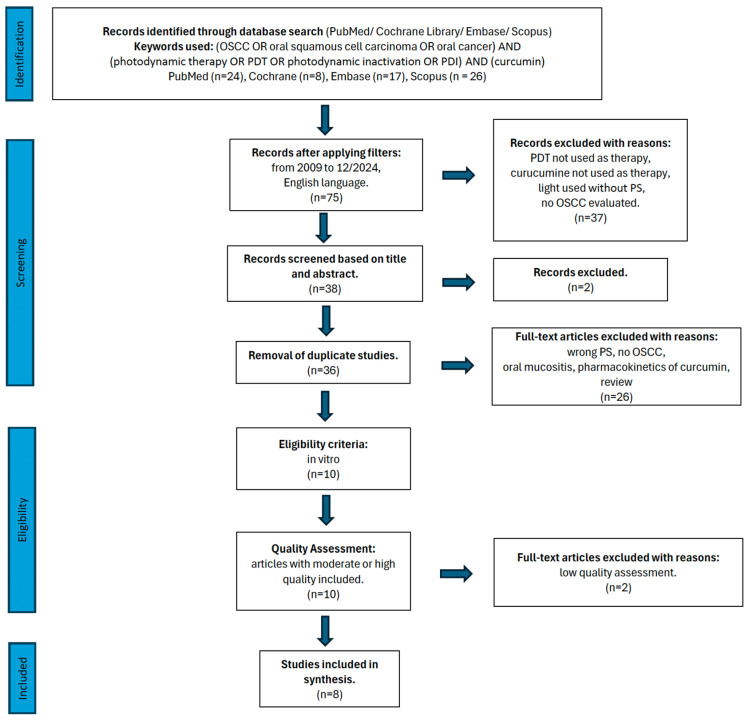
PRISMA 2020 flowchart showing selected criteria for included article reports.

A flowchart presenting the research approach in line with the PRISMA 2020 statement [22] is presented in Figure 1.

The initial database search identified 75 records. After restricting the results to English-language publications dated between 1 January 2009 and 31 December 2024, the pool was narrowed to 38 articles, which were then screened by title and abstract. Following this step, 37 articles were excluded for not meeting the inclusion criteria. After eliminating 2 duplicates, 38 unique articles proceeded to full-text evaluation. Of these, 25 were excluded based on predefined exclusion criteria. A detailed overview of the excluded studies and the corresponding reasons is provided in Table 2. The results of the risk of bias test are in Table 3.

### 3.3. Data Presentation

Data on the general characteristics of all nine studies that were ultimately included in the review were extracted. These studies met the eligibility criteria and included information on light sources and the properties of curcumin used as a photosensitizer in photodynamic therapy protocols.

### 3.4. General Characteristics of the Included Studies

A total of eight in vitro studies were included in the review. Table 4 lists the articles that met the inclusion criteria. Among them, eight were in vitro laboratory studies [2,4,46,47,49,50,51,53,56].

Eight studies investigated PDT effects on OSCC tumor cell suspensions [2,4,49,50,51,52,53,56]. Most in vitro assays were performed in 96-well plates [4,49,51,52,56], with one study using 24-well plates [50]. The cancer models included both established cell lines and primary patient-derived OSCC cultures. Four studies employed ATCC-standardized lines [4,50,51,53], while the others used cells directly isolated from patients [2,49,52,56]. One study also included cervical cancer and VX2 carcinoma cells from a New Zealand White rabbit [56]. Roschenko et al. [51] further distinguished between HPV-positive and HPV-negative OSCC cell lines.

### 3.5. Characteristics of Light Sources Used in PDT

The characteristics of the physical parameters of light sources meeting the inclusion criteria are presented in Table 5.

The included studies employed diverse light sources for photodynamic therapy. Three used LEDs with peak wavelengths of 457 nm [51,56] and 455 nm [50], delivering fluence levels between 1 and 8.6 J/cm^2^. Two studies applied diode lasers at 450 nm [2] and 650 nm [49]. Dual-source setups combining UVA (315–400 nm) and visible light (400–550 nm) were used in two studies [4,53]. One study utilized a broad-spectrum halogen lamp (400–700 nm) with a 200 mW output [52]. To ensure accuracy, two studies measured actual output with power meters to confirm alignment with device settings [2,52].

### 3.6. Characteristics of Curcumin Used as a Photosensitizer in PDT

The characteristics of curcumin used as a photosensitizer in photodynamic therapy in studies meeting the inclusion criteria are presented in Table 6.

Curcumin was the sole photosensitizer in seven studies [2,4,50,51,52,53,54,56], while one study compared it to chlorin e6 [49]. Six studies used free curcumin [2,4,50,51,52,53], and four incorporated it into nanocarriers or micelles [49,51,52,56]. Incubation times varied widely (20 min to 20 h), based on empirical testing or the prior literature. The most common durations were 1 h [2,4,52,53] and 4 h [49,52,56]. Photosensitizers were always stored in the dark before irradiation, and thorough mixing during incubation was ensured. In synthesizing results, greater weight was given to studies with low risk of bias based on predefined quality criteria, while findings from studies with moderate or high risk were interpreted cautiously due to limitations in reporting, controls, or outcome clarity.

## 4. Discussion

All included studies confirmed that curcumin-mediated photodynamic therapy (PDT) effectively reduced OSCC cell viability and proliferation. Ravera et al. found that free curcumin (1–10 µM) combined with 450 nm laser (15 J/cm^2^) induced cell cycle arrest and inhibited ATP synthesis and oxygen consumption [2]. Singh et al. showed ~70% cell death using curcumin-loaded silica nanoparticles (SiNp) with irradiation, versus ~20% for free curcumin [52]. Dujic et al. reported proliferation suppression in A431 cells using curcumin (0.25–2 µg/mL) with UVA or visible light [47]. Pavarina et al. observed an 87% metabolic activity reduction and necrotic changes with 20 µM curcumin and 455 nm LED [50]. Ambreen et al. showed curcumin liposomes with 457 nm light inhibited cancer cell migration, suggesting anti-metastatic effects [53,54,56]. Cell line sensitivity to curcumin-PDT varied by SCC origin and HPV status. Beyer et al. found HN OSCC cells were more responsive than HaCaT and A431 lines, with significantly reduced proliferation after treatment with 0.6–0.8 µg/mL curcumin and 400–550 nm light (1.65 J/cm^2^) [4]. Roschenko et al. demonstrated similar CUR-LCNP (curcumin-loaded lipid-coated nanoparticle) sensitivity across HPV-positive and HPV-negative HNSCC lines, with IC_50_ < 10 µmol/L, except for the cisplatin-resistant UT-SCC-26A line [51]. Notably, UT-SCC-26A showed the highest curcumin uptake, suggesting a survival mechanism despite high drug absorption. While no significant IC_50_ difference was observed between HPV status groups, the findings support CUR-LCNP-PDT as a promising option for treatment-resistant HNSCC [51]. The effectiveness of photodynamic therapy depends on many variable parameters, which can lead to significant discrepancies in results when different therapeutic protocols are used.

One key factor is the proper selection of the light wavelength. In PDT, the wavelength should closely match the maximum absorption of the photosensitizer. Curcumin’s maximum absorption occurs at 430 nm.. Beyer et al. observed differences in the efficiency of two light units: the effects induced by visible light (VIS) were stronger than those induced by UVA. This is consistent with curcumin’s maximum absorption; the VIS spectrum (380–780 nm) overlaps with this absorption peak, while UVA (315–400 nm) only partially activates curcumin, which absorbs in the 300 to 500 nm range [4]. This observation is further supported by the study by Dujic et al., where approximately 0.5 µg/mL of curcumin induced significant apoptosis when combined with visible light, but UVA had no effect at that concentration. However, higher concentrations of curcumin induced apoptosis with both visible light and UVA [53]. In all these studies, exposure of cancer cells to light alone, without treatment, did not affect cell viability. Incubation time, the interval between photosensitizer application and irradiation, is critical for optimal PDT efficacy. Ravera et al. found that a 1 h incubation with curcumin enhanced ATP synthesis inhibition in OHSU-974 cells after 450 nm laser exposure (15 J/cm^2^) [2]. Ambreen et al. reported no significant effect on viability with 2 h pre-incubation, suggesting at least 4 h is needed for effective uptake [20]. Singh et al. showed that longer incubation increased the dark toxicity of curcumin–SiNp nanoformulations: 4 h pre-treatment with 25 µM led to ~40% cell death without light and ~80% with light (12 J/cm^2^), whereas free curcumin showed no significant incubation-dependent effect [52].

Photosensitizer concentration is critical for PDT efficacy. Dujic et al. observed curcumin-induced inhibition of cancer cell proliferation starting at 0.25 µg/mL, with visible light (1 µg/mL) reducing proliferation to 17.3%—more effectively than UVA (31.1%)—while curcumin alone had no effect [53]. Ravera et al. reported a dose-dependent reduction in ATP synthesis with 1–10 µM curcumin, enhanced by light exposure [2]. Ambreen et al. showed curcumin liposomes had IC_50_ values of 9.52, 7.88, and 20.70 µmol/L for HeLa, UD-SCC-2, and Vx2 cells, respectively, under 3 J/cm^2^ irradiation [56]. Wu et al. similarly found higher curcumin concentrations decreased cell survival after 650 nm irradiation [49]. Pavarina et al. demonstrated significant metabolic reductions in HeLa cells—75.5%, 81.6%, and 87.5%—with 5, 10, and 20 µM curcumin under 455 nm LED (5.28 J/cm^2^), with dose-dependent dark toxicity at 10 and 20 µM (11–12% reduction) [50]. At 20 µM, no adherent cells remained, suggesting tumor necrosis. Beyer et al. confirmed dose-dependent PDT effects using LDH activity and DNA fragmentation as markers. LDH release increased with curcumin concentration (0.05–0.4 µg/mL) under UVA or VIS light, peaking at 0.4 µg/mL. DNA fragmentation increased up to 0.2 µg/mL, but declined at 0.4 µg/mL, indicating possible cytotoxic saturation or early necrosis [4].

The photodynamic efficacy of curcumin is strongly influenced by light parameters, particularly fluence. Ambreen et al. reported maximal phototoxicity at 5 J/cm^2^ but observed significant effects from 3 J/cm^2^ using a 457 nm LED [56]. Due to curcumin’s poor bioavailability, five studies utilized nanocarriers to enhance delivery [49,50,51,52,53,54,55,56,57,58,59]. Wu et al. found that CMCC (cancer cell membrane-coated nanoparticles) boosted curcumin uptake and phototoxicity via tumor-specific endocytosis [49]. Singh et al. demonstrated enhanced fluorescence, uptake, and cytotoxicity with curcumin–SiNp complexes, especially after longer incubation [52]. Nanocarriers also reduced dark toxicity—curcumin-loaded liposomes showed >90% cell viability without light, becoming cytotoxic only after irradiation [56]. Roschenko et al. confirmed effective delivery by lipid-coated nanoparticles in both HPV-positive and HPV-negative cell lines [51]. Curcumin exerts potent antiproliferative and pro-apoptotic effects at low concentrations [4,59,60]. OSCC lesions, often superficial, are ideal PDT targets given visible light’s shallow penetration [61,62], particularly in early-stage disease or when standard therapies are unsuitable [63]. Given rising HPV-positive OSCC rates, especially in young patients, curcumin-PDT may offer a safer alternative [64,65]. Notably, the cisplatin-resistant UT-SCC-26A cell line is responsive to curcumin-PDT [66], and PDT’s anti-metastatic effects further support its use [60,61,62,63,64,65,66,67]. Despite encouraging data, optimal curcumin dosing and light parameters remain undefined, and no clinical consensus exists [60,61,62,63,64,65,66,67,68,69,70,71]. Curcumin—derived from turmeric—modulates cancer-related pathways (inflammation, apoptosis, angiogenesis) and shows dose-dependent hormetic effects: antioxidant at low doses, autophagy induction at moderate doses, and apoptosis at high doses via ER/lysosomal disruption [72,73]. Although safe at up to 12 g/day (Phase I), clinical use is limited by low absorption and rapid metabolism. Strategies such as piperine co-administration, nanoformulations, and analogs (e.g., EF-24) are under development [74,75]. While curcumin is widely consumed and shows promise in cancer, cardiovascular, neurodegenerative, and inflammatory diseases, it remains unapproved for specific indications [76,77].

The included studies employed a variety of OSCC and related squamous cell carcinoma cell lines, which introduces a notable degree of heterogeneity in the experimental models. Differences in cellular origin, HPV status, and baseline sensitivity to photodynamic therapy likely influenced the outcomes. Although this heterogeneity reflects the biological diversity of OSCC, it complicates direct comparisons between studies and may affect the generalizability of the findings. One important limitation of this review is the inclusion of diverse cell lines, including both HPV-positive and HPV-negative subtypes, which may have differing intrinsic sensitivities to photodynamic therapy. This cellular heterogeneity adds variability to the pooled findings and should be considered when interpreting the results.

A key limitation of this systematic review is the high heterogeneity among the included studies. Variability was evident across several critical domains, including the type of cell lines used (e.g., different OSCC subtypes, HPV-positive vs. HPV-negative lines) and the use of a wide range of curcumin formulations such as free curcumin, liposomal curcumin, and silica-based nanoparticles. In addition, there were considerable differences in the light sources applied for photodynamic therapy, including diode lasers, LEDs, and halogen lamps, with varying wavelengths, fluence rates, and exposure times. Outcome measures also varied substantially, with some studies focusing on cell viability or apoptosis markers, while others assessed mitochondrial activity or DNA fragmentation. These methodological differences hinder direct comparability across studies and limit the strength of any overarching conclusions. Future studies should aim for greater standardization in PDT protocols and outcome assessments to enable more reliable cross-study comparisons and facilitate clinical translation. Additionally, there is a risk of publication bias, as studies with positive findings are more likely to be published in preclinical research. This bias may lead to an overestimation of the efficacy of curcumin-mediated photodynamic therapy and should be considered when interpreting the results of this review.

While the in vitro findings suggest that curcumin-mediated photodynamic therapy (PDT) holds promise for the treatment of oral squamous cell carcinoma, a significant translational gap remains between these experimental results and clinical application. In vitro models do not replicate the complex tumor microenvironment, including immune interactions, vascularization, and the influence of surrounding tissues. Moreover, the effective clinical translation of PDT in the oral cavity poses practical challenges such as the limited tissue penetration of visible light, especially in deeper or irregular tumor sites. The delivery of curcumin also remains a major obstacle due to its poor water solubility, rapid degradation, and limited bioavailability. Although nanocarrier-based formulations improve cellular uptake and phototoxicity in controlled settings, their pharmacokinetics, tissue distribution, and long-term safety must be validated in clinical trials. Furthermore, ensuring precise light delivery in anatomically complex regions of the oral cavity requires specially designed optical systems to achieve therapeutic fluence without damaging adjacent healthy tissues. Addressing these challenges is essential to bridge the gap between experimental efficacy and real-world therapeutic outcomes. Despite encouraging in vitro findings, there is a notable absence of clinical trials evaluating curcumin-mediated photodynamic therapy in patients with oral squamous cell carcinoma. Moreover, long-term safety and toxicity data specific to curcumin use in the oral cavity—particularly regarding mucosal integrity, systemic absorption, and repeated light exposure—are currently lacking, underscoring the need for rigorous clinical investigations.

This review has several limitations that affect interpretation. First, substantial methodological heterogeneity: differences in cell lines (HPV status, species), curcumin formulations (free, liposomal, nanoparticle), light parameters (wavelength, fluence, exposure time), and outcome measures—prevented direct comparisons and ruled out meta-analysis. Variability in incubation times and curcumin concentrations further hindered identification of optimal PDT conditions. Potential biases may influence findings. Publication bias is likely, and most studies lacked details on randomization, blinding, and replicates, increasing the risk of performance and detection bias. No studies reported adverse events or long-term toxicity, limiting safety assessment. In vitro models do not replicate the human tumor microenvironment, immune response, or pharmacokinetics, and no clinical trials have yet been conducted, highlighting a major translational gap. A broader systemic issue is the lack of standardized PDT protocols. No consensus exists on curcumin concentration, light settings, or delivery methods, limiting reproducibility and regulatory progress. Clinically, curcumin’s use as a photosensitizer introduces regulatory challenges. Although classified as GRAS for dietary use, therapeutic applications require strict evaluation of purity, stability, and GMP compliance. Light penetration limits, anatomical complexity of oral lesions, and the need for specialized equipment also pose practical barriers. Future studies should adopt standardized protocols, test clinically relevant models, and initiate early-phase trials to assess safety, efficacy, and delivery strategies. Regulatory and technical hurdles must be addressed to enable clinical translation of curcumin-PDT for OSCC.

## 5. Conclusions

This systematic review confirms that curcumin-mediated photodynamic therapy (PDT) reduces OSCC cell viability, induces apoptosis, and disrupts metabolic activity in vitro. Enhanced effects were observed with nanoparticle-based delivery systems and light sources around 430–457 nm. Despite methodological heterogeneity, curcumin consistently demonstrated low dark toxicity and strong photodynamic efficacy. However, the lack of standardized PDT protocols and absence of in vivo or clinical studies limit clinical translation. Future research should focus on harmonizing treatment parameters and initiating preclinical trials to evaluate bioavailability, safety, and efficacy in complex biological systems.

## Figures and Tables

**Table 1:** Selection criteria for papers included in the systematic review.

Inclusion Criteria	Exclusion Criteria
In vitro studies using oral squamous cell carcinoma cells. Use of free curcumin or nanoformulated curcumin, specifically intended as a photosensitizer (not solely as a chemotherapeutic agent). The method of eliminating cancer cells used in in vitro studies was curcumin-mediated photodynamic therapy. Original, peer-reviewed in vitro research articles. Published in English. Studies published between 1 January 2009 and 31 December 2024.	Editorial correspondence or commentary (e.g., letters to the editor) Non-contemporary literature or historical analyses Secondary research articles, including literature and systematic reviews Non-peer-reviewed sources such as books, reports, or policy documents Redundant articles or those sharing identical ethical clearance identifiers Articles not published in the English language Studies where photodynamic therapy was not implemented as a treatment modality Investigations in which curcumin was not applied as the active photosensitizer Research employing a photosensitizer other than curcumin Use of light therapy in the absence of any photosensitizing agent Studies not involving oral malignancies as a target condition

**Table 2:** List of excluded studies and justification for their exclusion.

Reason for Exclusion	Reference Number
review of the structure and pharmacokinetics of curcumin	[15,18,24]
chemo-photothermal therapy; human lung and liver cancer cells	[25,26,27]
oral mucositis in patients with cancer	[28,29,30,31,32]
breast cancer	[33]
no photodynamic therapy	[34,35,36,37,38,39]
ALA-PDT	[40]
review; PDT of OSCC	[41,42]
osteosarcoma	[43]
pharmacokinetics of two-photon active nucleus-targeting carbon dots	[44]
review; therapeutic role in cancer	[45]
no access	[46]
ovarian adenocarcinoma	[47]
no OSCC	[48]

ALA-PDT—5-aminolevulinic acid photodynamic therapy; OSCC—oral squamous cell carcinoma; PDT—photodynamic therapy.

**Table 3:** Risk of bias performed on studies that met the inclusion criteria.

Reference	[PS]	Origin of PS	Incubation Time	Light Source Parameters	Powermeter	Clinical Cancer Cell Lines	Negative Control Group	Numerical Results Available (Statistics)	No Missing Outcome Data	Total Score
[2]	yes	no	yes	yes	yes	yes	yes	yes	yes	8
[4]	yes	yes	yes	no	no	no	yes	yes	no	5
[39]	yes	yes	yes	yes	no	yes	yes	yes	yes	8
[49]	yes	yes	yes	yes	no	yes	yes	yes	yes	7
[50]	yes	yes	yes	yes	no	no	yes	yes	yes	7
[51]	yes	yes	yes	yes	no	no	yes	yes	yes	7
[52]	yes	no	yes	yes	yes	yes	yes	yes	no	7
[53]	yes	yes	yes	no	no	no	yes	yes	no	5
[54]	yes	no	yes	no	no	no	yes	yes	no	4
[55]	no	no	yes	no	no	no	yes	yes	no	3

PS—photosensitizer.

**Table 4:** General characteristics of studies that met the eligibility criteria.

Author/Year	Reference Number/Country/Year of Publication	Study Design	Cell Culture	Study Group	Outcomes
Ravera et al. (2024) [2]	[2] Italy South Africa 2024	In vitro study	To eliminate potential bias associated with the FANC-A gene mutation, the OHSU-974 cell line (originally obtained from Cincinnati Children’s Hospital Medical Center, Cincinnati, OH, USA) was genetically corrected using the S11FAIN retroviral vector.	control untreated, laser, curcumin, curcumin + laser, lasered curcumin *n* = 3	Curcumin shows an ability to reduce the aerobic energy metabolism function of HNSCC at concentrations of 1 and 10 μM. The anti-tumor activity of curcumin was further enhanced by a 60 s exposure to 15 J/cm^2^ of 450 nm laser light.
Beyer et al. (2017) [4]	[4] Germany Spain 2017	In vitro study on 96-well microtiter plates with a density of approximately 2 × 10^4^ cells/0.33 cm^2^	The study utilized three human cell lines: the oral squamous cell carcinoma line HN ACC 417 (DSMZ, Leipzig, Germany), the spontaneously immortalized human keratinocyte line HaCaT 26, and the human epidermoid carcinoma line A431 (ATCC CRL-1555™).	control unirradiated 0 h, control unirradiated 16 h, control unirradiated 1 J/cm^2^ UVA 16 h, control unirradiated 5 min VIS 16 h; CUR 0.01–1 µg/mL	In cultures treated with curcumin concentrations of 0.8 µg/mL and 1 µg/mL in combination with UVA as well as in cultures treated with 0.6 µg/mL and 0.8 µg/mL curcumin in combination with VIS cell retraction and dynamic plasma membrane blebbing were evident. Lactate dehydrogenase activity in supernatants increased after UVA irradiation when applying 0.4 µg/mL–1 µg/mL curcumin and 0.4 µg/mL curcumin after VIS irradiation. DNA fragmentation of cell cultures treated with curcumin and light was significantly increased.
Ambreen et al. (2020) [56]	[56] Germany Pakistan 2020	In vitro study on 96-well transparent microtiter plate at ~1 × 10^4^ (HeLa and VX2) or ~6 × 10^4^ (UD-SCC-2) cells per well (0.35 cm^2^)	The HeLa cell line, derived from cervical cancer, and the UD-SCC-2 cell line, originating from head and neck squamous cell carcinoma (HNSCC) at the University of Düsseldorf, Germany, were used. Additionally, fresh VX2 cells were obtained from a VX2 carcinoma in a New Zealand White (NZW) rabbit.	Control (Dark); 1/3/5 J/cm^2^; Curcumin Liposomes/DMSO 0–100 umol/L *n* = 3	Curcumin liposomes were capable of generating a PDT-triggered response in three papilloma virus-associated tumor cell lines, leading to major cell death.
Wu et al. (2023) [49]	[49] China 2023	In vitro study in 96-well plates at a density of 1 × 10^4^ cell	The CAL-27 tongue cancer cell line was donated to the laboratory of Shanghai Ninth People’s Hospital.	CMCC+L, CMCC-L, CC+L, CMC+L, MCC+L; Ce6: 5, 2.5, 1.25 µg/mL; Cur: 2, 1, 0.5 µg/mL (CMCC—cancer cell membrane coated mesoporous silica nanoparticles loaded with Ce6/Cur, MCC—mesoporous silica nanoparticles loaded with Ce6 and Cur, CMC—cell membrane coated mesoporous silica nanoparticles loaded with Ce6, CC—Ce6 + Cur, Ce6—chlorin e6)	The biomimetic nanoplatform apparently enhanced the therapeutic effect of PDT by curcumin disturbing the ROS-defense system.
Pavarina et al. (2012) [50]	[50] Brazil 2012	In vitro study on 24-well plates	The immortalized Hela cell line, purchased from Adolfo Lutz Institute (São Paulo, SP, Brazil)	C+L+, C+L-, C-L+, C-L- *n* = 5	The association of CUR and light achieved a significant reduction in cell metabolism of 87.3%.
Roschenko et al. (2023) [51]	[51] Germany 2023	In vitro study on 96-well plates 15,000 cells/0.35 cm^2^ (per well)	The cell lines UM-SCC-47, UPCI-SCC-154, and UM-SCC-104 represent the HPV-positive subgroup, while UM-SCC-3, UM-SCC-27, and UT-SCC-26A belong to the HPV-negative subgroup. These lines were sourced from the University of Michigan (USA), the University of Pennsylvania (USA), and the University of Turku (Finland).	CUR-LCNPs+L+, Free CUR+L+, CUR-LCNPs+L-, Free CUR+L- *n* = 3	The photodynamic efficacy of CUR-LCNPs was evident in inhibiting cell viability across a total of six HNSCC cell lines, both HPVpos and HPVneg, with only low dark toxicity.
Singh et al. (2014) [52]	[52] India 2014	In vitro study on 96-well plate 5 × 10^4^ 4451 cells	The human squamous cell carcinoma (4451) cell line, derived from an oral cavity carcinoma, was established at the Institute of Nuclear Medicine and Allied Sciences (INMAS) in Delhi, India.	control, Cur, Cur + 12 J/cm^2^, Cur + 20 J/cm^2^ (1 h/4 h) (Cru free/Cur-SiNp)	Curcumin–SiNp formulation enhances uptake and cytotoxic effects of curcumin in oral cancer cells. Photodynamic activity of curcumin in nanoformulation is enhanced as indicated by an increase in cell killing and by inhibition of NF-κB activity
Dujic et al. (2009) [53]	[53] Germany 2009	In vitro study on microwell plates at a density of 2 × 10^4^ cells per 0.33 cm^2^	The A431 cell line, a human epidermoid carcinoma model, was obtained from the American Type Culture Collection (ATCC).	control/light-protected/UVA/VIS, 0/0.25/0.5/1/2 µg/mL *n* = 8	A combination of light and curcumin amplifies the anti-growth and proapoptotic effects of curcumin in a tumor model. The dose of visible light showed stronger effects than 1 J/cm^2^ UVA when applied with equal amounts of curcumin.

CUR—curcumin; CMCC—cancer cell membrane coated mesoporous silica nanoparticles loaded with Ce6/Cur; MCC—mesoporous silica nanoparticles loaded with Ce6 and Cur; CMC—cell membrane coated mesoporous silica nanoparticles loaded with Ce6; CC—Ce6 + Cur; Ce6—chlorin e6; SiNp—organically modified silica nanoparticles; DMSO—dimethyl sulfoxide; L—light; C—curcumin; CUR-LCNPs—lipid-coated polymer nanoparticles encapsulating curcumin.

**Table 5:** Physical parameters of light sources derived from studies meeting the eligibility criteria.

Author/Year	Light Source	Wavelength (nm)	Energy Density (Fluence) (J/cm^2^)	Power Output (mW)	Irradiation Time (s)
Ravera et al. (2024) [2]	Diode laser (Garda Laser snc, Verona, Italy)	450	15	250	60
Beyer et al. (2017) [4]	UVA, Waldmann, Villingen-Schwenningen, Germany; VIS, 5500 lx, Philips GmbH, Hamburg, Germany	315–400 (UVA) 400–550 (VIS)	1 (UVA) 1.65 (VIS)		300
Ambreen et al. (2020) [56]	A low-power LED device (Lumundus GmbH, Eisenach, Germany) was equipped with two different LEDs of 457 nm (blue) and 652 nm (red) wavelengths.	457	1, 3, 5	220.2 W/m^2^	45, 136, 227
Wu et al. (2023) [49]	Laser	650	20 mW/cm^2^		300
Pavarina et al. (2012) [50]	A light emitting diode (LED) based device, composed of eight royal blue LEDs (LXHL-PR09, Luxeon^®^ III Emitter, Lumileds Lighting, San Jose, CA, USA)	455	5.28	22 mW/cm^2^	240
Roschenko et al. (2023) [51]	A prototype low-power light-emitting diode (LED) device designed to fit multiwell plates (Lumundus GmbH, Eisenach, Germany)	457	8.6	100 mA	390
Singh et al. (2014) [52]	Halogen lamp equipped with a multimode fiber (Applied Optical Technologies, Plot No. 147, Rd Number 24, Wagle Industrial Estate, Thane West, Thane, Maharashtra 400604, India)	400–700	12, 20	200	
Dujic et al. (2009) [53]	UV therapy system UV 3003 K, Waldmann, Villingen-Schwenningen, Germany; 5500 lx visible light (10x 40 W lamps, distance 45 cm, Philips GmbH, Hamburg, Germany)	315–400 (UVA) 400–550 (VIS)	1 (UVA) 1.65 (VIS)		300

LED—light-emitting diode.

**Table 6:** Characterization of curcumin used as PS in studies meeting the eligibility criteria.

Author/Year	Incubation Time (in Minutes)	The Way of Presentation of Curcumin	Concentration/s of PS Used
Ravera et al. (2024) [2]	60	Curcumin dissolved in DMSO	0.1, 1, 10 µM
Beyer et al. (2017) [4]	60	Curcumin (Sigma-Aldrich Taufkirchen, Eschenstrasse 5, 82024 Taufkirchen, Germany.)	0.01 μg/mL to 1 μg/mL
Ambreen et al. (2020) [56]	240	Curcumin (Sigma-Aldrich Taufkirchen, Germany) Curcumin liposomes	IC50 at 3 J·cm^−2^: 9.52 µmol/L for HeLa, 7.88 µmol/L for UD-SCC-2, and 20.70 µmol/L for VX2 cells
Wu et al. (2023) [49]	240	Curcumin (Sigma-Aldrich, Shanghai) was dissolved in DMSO to 20 mg/mL solution. Synthesis of mesoporous silica nanoparticles loaded with curcumin	0.5, 1, 2 µg/mL
Pavarina et al. (2012) [50]	20	Curcumin (Sigma Aldrich, St. Louis, MO, USA); A stock solution of CUR (200 µM) was prepared in 10% DMSO and then diluted in saline solution to obtain the concentrations to be tested	5, 10, 20 µM
Roschenko et al. (2023) [51]	240	Curcumin (Alfa Aesar, Kandel, Germany); CUR-LCNP	0, 1, 5, 10, 20, 30, 40, 50 µM/L
Singh et al. (2014) [52]	1, 2, 4 and 20	Curcumin dissolved in DMSO and curcumin-SiNp complex	10, 25 µM
Dujic et al. (2009) [53]	60	30 mg curcumin (Sigma, Deisenhofen, Germany) was dissolved in 1 mL DMSO	0.25 to 2 µg/mL

CUR—curcumin, DMSO—dimethyl sulfoxide, CUR-LCNP—lipid-coated polymer nanoparticles encapsulating curcumin.
